# Examining Assumptions about Female Dominance in Autoimmune Disease

**DOI:** 10.1289/ehp.120-a107

**Published:** 2012-03-01

**Authors:** Bob Weinhold

**Affiliations:** Bob Weinhold, MA, has covered environmental health issues for numerous outlets since 1996. He is a member of the Society of Environmental Journalists.

Autoimmune diseases are generally increasing in the United States and many other countries, for poorly understood reasons, affecting tens of millions of people.^1^ Environmental factors are among the suspects behind these diseases, because genetic factors alone can’t explain the significant rise in recent decades of diseases such as multiple sclerosis, Crohn disease, systemic lupus erythematosus, and type 1 diabetes. One common assumption that many experts have adopted, based on evidence pieced together over the decades, is that females are affected more than males by autoimmune diseases.^1^^,^^2^^,^^3^ But the key assumption that females are more susceptible to autoimmune diseases may be wrong in certain circumstances, says J. Michael Pollard, an associate professor of molecular medicine at the Scripps Research Institute in La Jolla, California.

A number of autoimmune diseases do have a female predominance overall, Pollard says. However, after reviewing selected studies of autoimmune diseases with links to any of more than a dozen known or suspected environmental factors, he found that the presumed female predominance in many autoimmune disorders may be correct in fewer instances than thought; the link is unclear in other situations and may simply not exist in still others.^4^ Pollard does not disagree with the sex bias found for idiopathic autoimmune diseases (those for which a cause is unknown). “The question addressed in my review was, is the . . . bias in idiopathic autoimmune disease also reflected in studies in which an associated environmental exposure has been studied?” he says.

One reason for the potentially misleading evidence could be confounding factors such as sex-biased activities. For instance, Pollard found that some evidence reflects the female predominance for an exposure source, as with certain cosmetics and the diseases lupus and rheumatoid arthritis. In a reverse example, he found that the apparent male predominance of several autoimmune diseases resulting from silica exposure could reflect males’ dominating the occupations where this exposure tends to occur (e.g., mining and masonry). That became clearer after he identified a study of female subjects that found a relative risk for rheumatoid arthritis from silica exposure even greater than the composite average for males as reported in a meta-analysis of 10 studies.

In other studies, Pollard found mixed evidence for predominance by one sex or the other, with results depending on the details. For instance, for lupus caused by prescription drugs, females are more vulnerable when using procainamide (a heart medication), but use of chlorpromazine (an antipsychotic) is linked more strongly in males.

Human autoimmunity studies often lack corresponding animal studies, because many of the latter adopt the assumption of female predominance and test only females, Pollard says. But studies of the same autoimmune disorder in different strains of animals have shown very different results for which sex, if any, predominates. This suggests to Pollard that more studies that include male animals might lead to different results regarding one sex’s predominance for environmentally induced autoimmune disease.

As many as 50 million Americans may suffer from autoimmune diseases, which affect neurological, musculoskeletal, connective tissue, gastrointestinal, cardiovascular, endocrine, vision, skin, and other systems.^1^ There are more than 80 recognized autoimmune diseases, and many more diseases are suspected of having an autoimmune component.^1^ Environmental factors suspected of being involved in the development of autoimmune diseases include prescription drugs, silica, solvents, cosmetics, smoking, infectious agents, and ionizing and ultraviolet radiation.^4^

In the rare situations where there is overlap in human and animal studies evaluating both sexes for the same autoimmune disease following exposure to the same environmental substance, the animal and human data sometimes coincide. This was the case for silica and lupus, for which studies showed no sex predominance.

But studies concurrently addressing all these factors are extremely rare, says Noel Rose, director of the Johns Hopkins Center for Autoimmune Disease Research. Only 2 others not reviewed by Pollard immediately came to mind—links between excess dietary iodine and autoimmune thyroiditis^5^ and between certain strains of *Campylobacter* bacteria and Guillain-Barré syndrome.^6^ This shortage of comprehensive research means it’s very difficult to understand key factors such as whether an environmental agent is causing an autoimmune disease, is accelerating a preexisting condition, or even might remedy a budding problem, he says.

One technique that could be used to improve understanding of the animal data and to help determine specific mechanisms in humans is to also expose feminized males and masculinized females^7^ to an environmental substance, says Frederick Miller, acting clinical director and chief of the Environmental Autoimmunity Group at the National Institute of Environmental Health Sciences. That would help researchers sort out whether sex-specific genes or sex hormone effects are affecting the outcome of the tested environmental substance.

But selecting which substances to investigate remains difficult. Miller says a rigorous national registry of autoimmune disease cases is needed to aid this effort. “Such a registry, combined with carefully conducted environmental epidemiology studies, would greatly help us home in on the suspects most likely to be triggering autoimmune diseases,” he says.

Rose, who found the Pollard study to be generally strong and meticulous, agrees with Miller’s point. “The genetic research is really progressing,” he says. “The part that is really stalled is the environmental side. That starts with epidemiology,” where studies to date have rarely been well controlled or of adequate size. Pollard says filling that critical void could at least improve the odds of resolving sex-related differences and other autoimmune puzzles. “Populations of patients with idiopathic disease are readily available,” he says. “What is much harder and severely lacking are populations with an appropriate environmental exposure that can be studied for evidence of autoimmune disease. It really is very frustrating not having the appropriate human populations to study.”
